# Human iPSC-derived cardiomyocytes cultured in 3D engineered heart tissue show physiological upstroke velocity and sodium current density

**DOI:** 10.1038/s41598-017-05600-w

**Published:** 2017-07-14

**Authors:** Marc D. Lemoine, Ingra Mannhardt, Kaja Breckwoldt, Maksymilian Prondzynski, Frederik Flenner, Bärbel Ulmer, Marc N. Hirt, Christiane Neuber, András Horváth, Benjamin Kloth, Hermann Reichenspurner, Stephan Willems, Arne Hansen, Thomas Eschenhagen, Torsten Christ

**Affiliations:** 1 Department of Cardiology-Electrophysiology, University Heart Center, Hamburg, Germany; 2 Department of Experimental Pharmacology and Toxicology, University Medical Center Hamburg-Eppendorf, Hamburg, Germany; 3 DZHK (German Center for Cardiovascular Research), partner site Hamburg/Kiel/Lübeck, Hamburg, Germany; 4 Department of Pharmacology and Pharmacotherapy, Faculty of Medicine, University of Szeged, Szeged, Hungary; 5 Department of Cardiovascular Surgery, University Heart Center, Hamburg, Germany

## Abstract

Human induced pluripotent stem cell-derived cardiomyocytes (hiPSC-CM) are a promising tool for drug testing and modelling genetic disorders. Abnormally low upstroke velocity is a current limitation. Here we investigated the use of 3D engineered heart tissue (EHT) as a culture method with greater resemblance to human heart tissue in comparison to standard technique of 2D monolayer (ML) format. I_Na_ was measured in ML or EHT using the standard patch-clamp technique. I_Na_ density was ~1.8 fold larger in EHT (−18.5 ± 1.9 pA/pF; n = 17) than in ML (−10.3 ± 1.2 pA/pF; n = 23; p < 0.001), approaching densities reported for human CM. Inactivation kinetics, voltage dependency of steady-state inactivation and activation of I_Na_ did not differ between EHT and ML and were similar to previously reported values for human CM. Action potential recordings with sharp microelectrodes showed similar upstroke velocities in EHT (219 ± 15 V/s, n = 13) and human left ventricle tissue (LV, 253 ± 7 V/s, n = 25). EHT showed a greater resemblance to LV in CM morphology and subcellular Na_V_1.5 distribution. I_Na_ in hiPSC-CM showed similar biophysical properties as in human CM. The EHT format promotes I_Na_ density and action potential upstroke velocity of hiPSC-CM towards adult values, indicating its usefulness as a model for excitability of human cardiac tissue.

## Introduction

Animal-heart tissue is commonly used as a model for human-heart tissue, but exhibits a significantly different action potential (AP) duration and shape, due to different ion channel contributions, interactions and regulation. Human induced pluripotent stem cell-derived cardiomyocytes (hiPSC-CM) have the great advantage of generating human-like AP-duration and shape. In addition, hiPSC-CM represent a theoretically unlimited source of CM, lacking the ethical concerns that come along with sacrificing animals. The recent progress in the development and generation of hiPSC provides great opportunities to study individualised cardiac electrophysiology, focusing on genetic disorders^1^ and individualised drug treatment^2^. However, there are concerns about the immaturity of hiPSC-CM^3^. One important difference relates to AP upstroke-velocity which, in initial publications, was found to be markedly lower (~2–50%) in hiPSC-CM^4–6^ than in adult CM^7^. These findings suggest lower sodium current (I_Na_) density in hiPSC-CM, which is of great physiological importance as I_Na_ determines excitability, conductance velocity, refractoriness and triggered activity. Furthermore, I_Na_ is an established drug target for antiarrhythmic therapy (flecainide, propafenone, amiodarone, vernakalant and ranolazine). Recent improvements have been brought about by co-culture with non-cardiac cells^8^, long-term culture^9^, hormone stimulation^1^, continuous field stimulation^10^ and variation of substrate stiffness^11, 12^, revealing upstroke velocities of up to 147 V/s^12^. While these values approach the expected range (200–300 V/s) for human adult ventricular tissue, differences remain and a head-to-head comparison under same conditions is lacking.

An alternative approach to increase the maturation of hiPSC-CM is cardiac tissue engineering^13^. CM in hydrogel-based engineered heart tissue (EHT) form a synchronously beating syncytium, which generates contractile force by rhythmically deflecting the two elastic silicone posts it is attached to and thereby performs auxotonic contractile work^14, 15^. Morphological and functional evidence suggest that hiPSC-CM reach a higher degree of maturity in EHT^15^, but electrophysiological data are lacking. Here we directly compared upstroke velocity in hiPSC-CM cultured in 3D (EHT) and in human heart tissue biopsies obtained during the implantation of left ventricular assist devices (LVAD) or heart transplantation, and studied I_Na_ properties in hiPSC-CM from 2D monolayers (ML) and EHT under the conditions published for human adult CM.

## Results

### Cell capacitance and sodium current

**Figure 1:**
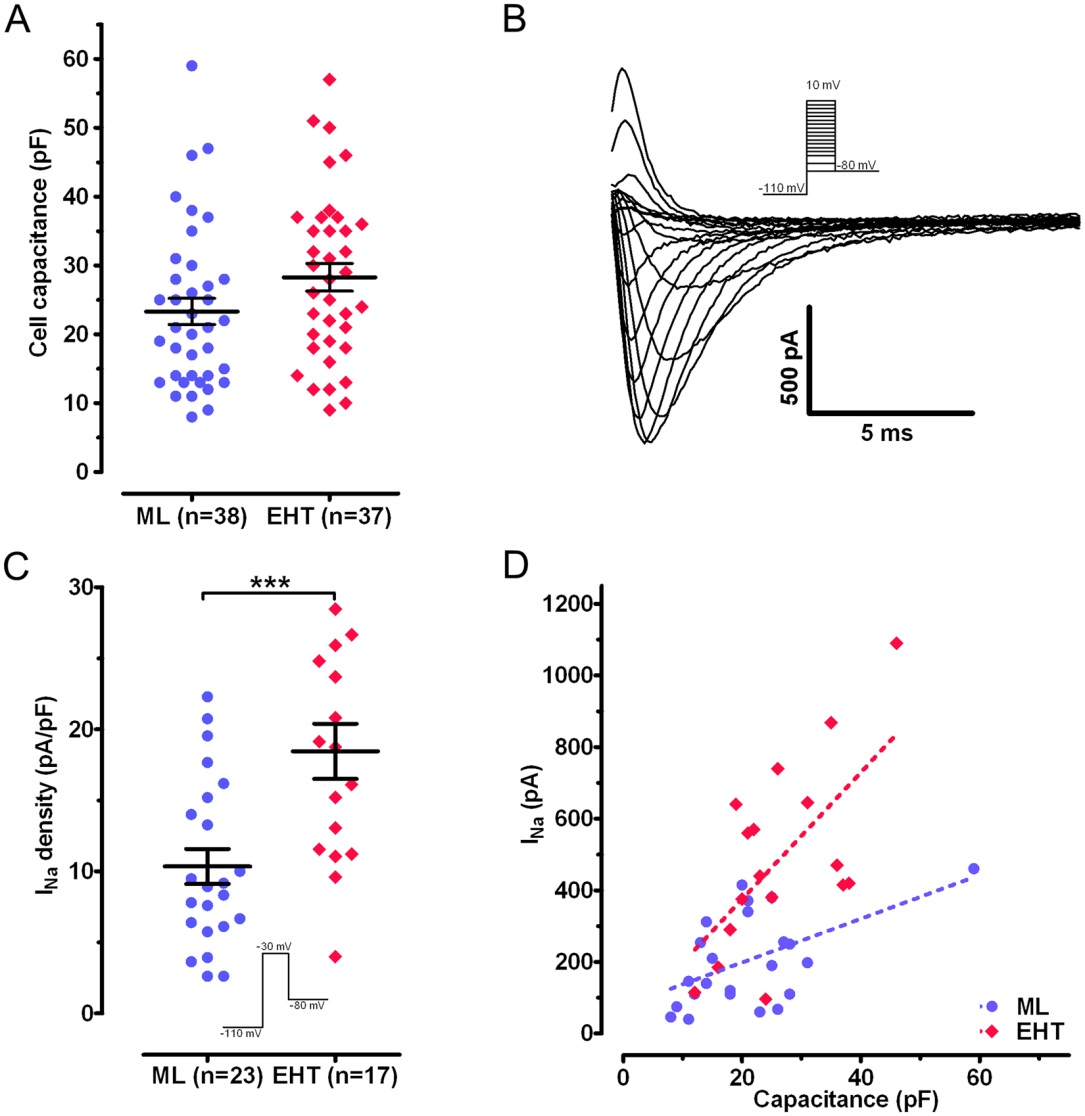
Cell capacitance and sodium current. (**A**) Scatter plot of cell capacitance in hiPSC-CM (mean values in Table 1). (**B**) Family of original Na current traces elicited by the protocol shown in the inset. (**C**) Scatter plot of I_Na_ density in hiPSC-CM for voltage-clamp pulse to −30 mV from a holding potential of −110 mV (EHT vs ML: ***p < 0.001). (**D**): Correlation of I_Na_ amplitude and cell capacitance. Best fit values for slope: ML 9.7 ± 2.4 pA/pF vs. EHT 16.9 ± 5.9 pA/pF. Deviation from zero slope was significant for ML (p < 0.001) and EHT (p < 0.05).

**Figure 2:**
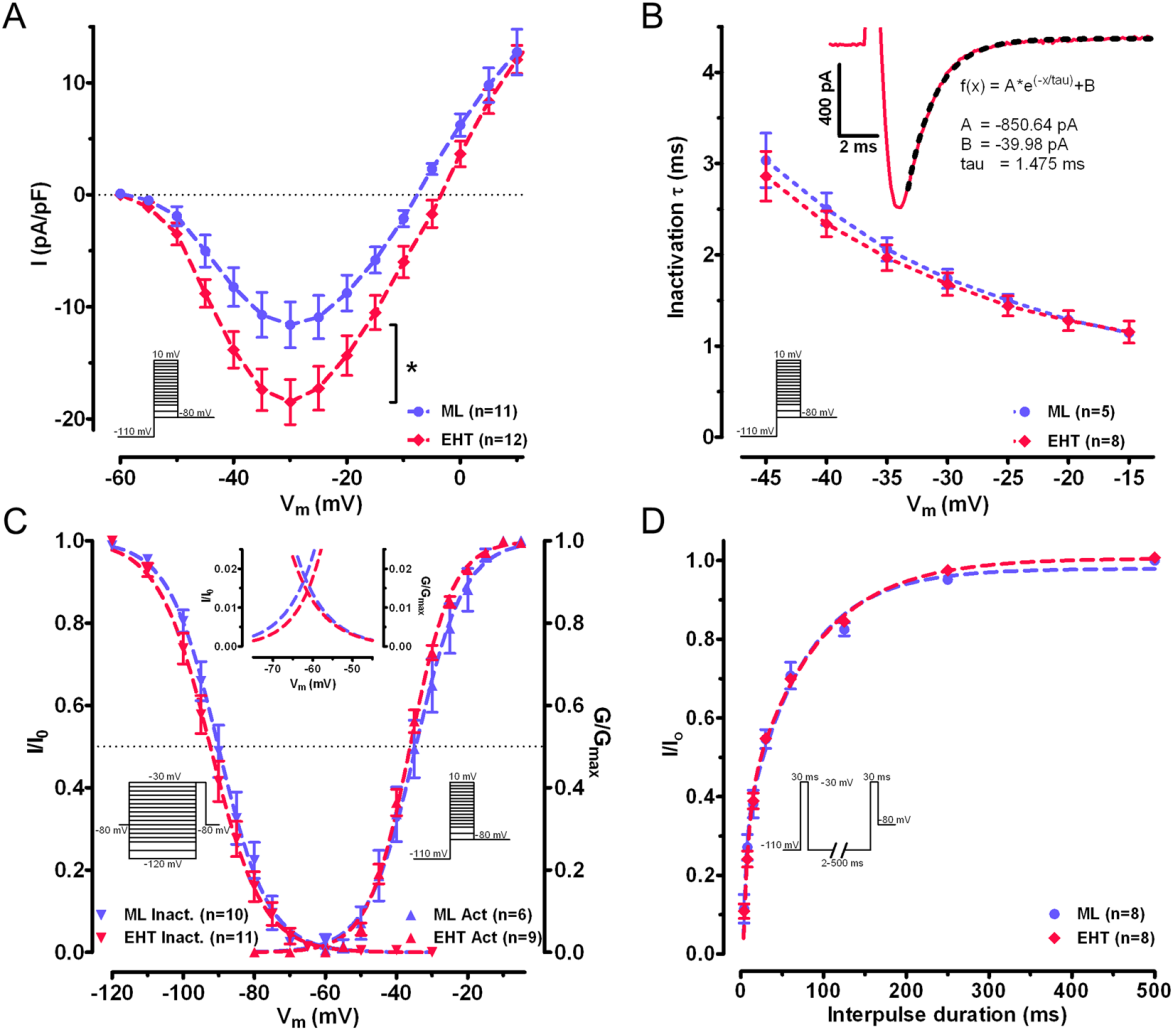
Biophysical properties of sodium current. (A) Current–voltage relationship in human induced pluripotent stem cell-derived cardiomyocytes: engineered heart tissue vs. monolayer, *p < 0.05. (**B**) Inactivation kinetics of I_Na_ were fitted by a single exponential function and characterised by the time constant τ as a function of the depolarisation step. An exemplary original trace of I_Na_ in EHT hiPSC-CM is shown (inset) with a function fitted as a dotted line and corresponding parameters. (**C**) Steady-state inactivation and activation relations for I_Na_. The dotted lines represent fitted data calculated by a Boltzmann function. Insets show curves at higher magnification to illustrate the overlap at ~62.5 mV and maximal window current of 1–2%. (**D**) Recovery from inactivation of I_Na_ using a double-pulse protocol varying intervals (2 to 500 ms). Dotted line represents curve fits by a two-phase exponential function.

We compared the I_Na_ of EHT with standard 2D ML using the whole-cell patch clamp technique. Cell size as measured by the cell capacitance showed no statistically significant difference between EHT and ML (Fig. 1A): EHT 28.2 ± 2.0 pF (n = 37) vs. ML 23.3 ± 1.9 pF (n = 38). We studied I_Na_ at a reduced extracellular Na concentration, in order to ensure good voltage control and comparability to previous studies on human adult CM^7, 16, 17^. As expected, current amplitude showed a proportional relation to cell size (Fig. 1D). Mean I_Na_ density was remarkably higher (~80%) in EHT (−18.5 ± 1.9 pA/pF; n = 17) than in ML (−10.3 ± 1.2 pA/pF; n = 23; p < 0.001; Fig. 1C). I–V curves (Fig. 2A) show that the I_Na_ was activated around −55 mV and peaked at −30 mV in EHT and ML. EHT hiPSC-CM showed higher I_Na_ density than ML over the entire activation range (p < 0.05, Fig. 2A). Thus, EHT showed increased I_Na_ density in comparison to ML. To test for a late sodium current, we measured currents at the end of our test-pulse. Currents amounted to −68.9 ± 13.9 pA under control condition and to −70.1 ± 13.9 pA after the application of 30 µM tetrodotoxin (TTX; n = 10, ns, paired t-test). Therefore, we did not find evidence for a persistent/late I_Na_.

### Inactivation, activation and recovery from inactivation

The contribution of I_Na_ to the electrical activity of CM depends critically on the voltage and time-dependent activation and inactivation. Activation curves were calculated from individual I–V curves by normalising peak current amplitudes for their actual driving force and a Boltzmann function was fitted to the data set (Fig. 2C). Voltages for the half-maximum activation (V_0.5act_) of I_Na_ and curve steepness (k_act_) did not differ between EHT and ML (Table 1). In order to characterise the inactivation kinetics of I_Na_, we fitted a single exponential function to current traces at different test pulse potentials (Fig. 2B). Time constants were shorter at increasing voltages of the test pulse without differences between EHT and ML.

**Table 1:** Biophysical parameters of ML and EHT cultured hiPSC-CM. HiPSC-CM: human induced pluripotent stem cell-derived cardiomyocytes; n: number of cardiomyocytes; I_Na_ density measured at −30 mV from −110 mV holding potential; V_0.5_: voltage of half-maximal (in)activation; k: slope factor of voltage-dependence of (in)activation; τ_fast_ /τ_slow_: fast and slow time constants of recovery from inactivation. Values are mean ± SEM.

	ML	n	EHT	n	p-value
**Cell capacitance** (pF)	23.3 ± 1.9	38	28.2 ± 2.0	37	0.081
**I**_**Na**_ **density** (pA/pF) at −30 mV test pulse	−10.3 ± 1.2	23	−18.5 ± 1.9	17	<0.001
**Inactivation** τ (ms) at −30 mV test pulse	1.68 ± 0.1	5	1.73 ± 0.1	9	0.758
**Activation**		6		9	
V_0.5_ (mV)	−34.6 ± 2.1		−36.2 ± 0.7		0.353
k_act_	5.8 ± 0.2		6.2 ± 0.2		0.187
**Steady-state inactivation**		10		11	
V_0.5_ (mV)	−89.8 ± 1.6		−91.3 ± 1.3		0.493
k_inact_	6.1 ± 0.5		6.7 ± 0.2		0.252
**Recovery of inactivation**		8		8	
Proportion fast (%)	54.7 ± 14.1		49.7 ± 6.3		0.376
**τ**_**fast**_ (=1/k_fast_) (ms)	5.4 ± 1.3		6.7 ± 1.3		0.488
**τ**_**slow**_ (=1/k_slow_) (ms)	93.4 ± 17.3		90.4 ± 9.0		0.857

In general, resting membrane potential (RMP) in CM is less negative than V_0.5inact_^17^. Therefore, only a minority of cardiac Na channels can be activated. Even small changes in RMP have strong effects on Na channel availability. We simulated different RMPs by applying variable conditioning pre-pulses from −120 mV to −30 mV for 1000 ms to determine steady-state inactivation. The mean data revealed no differences between EHT and ML in V_0.5inact_ or k_inact_ (Table 1). A Boltzmann curve (Fig. 2C) showed that activation and steady-state inactivation curves overlap between ~−77 mV and ~−45 mV (inset Fig. 2C). The maximal overlap was reached at −61.9 mV (ML) and −61.3 mV (EHT), where normalised I_Na_ availability and conductance were 1.4% (EHT) and 1.8% (ML) of the maximum. EHT and ML did not differ in window current amplitude or in voltage-dependency.

The refractoriness of heart muscle depends critically on the fast recovery of I_Na_ from inactivation. The proportion of second I_Na_ to first I_Na_ was decreased by shorter interpulse duration as shown by the mean data and the two-phase exponential fit (Fig. 2D). Fitting a two-phase exponential function to the data set of each individual cell revealed no difference between EHT and ML (Table 1).

### Tetrodotoxin sensitivity and expression of sodium channel isoforms

**Figure 3:**
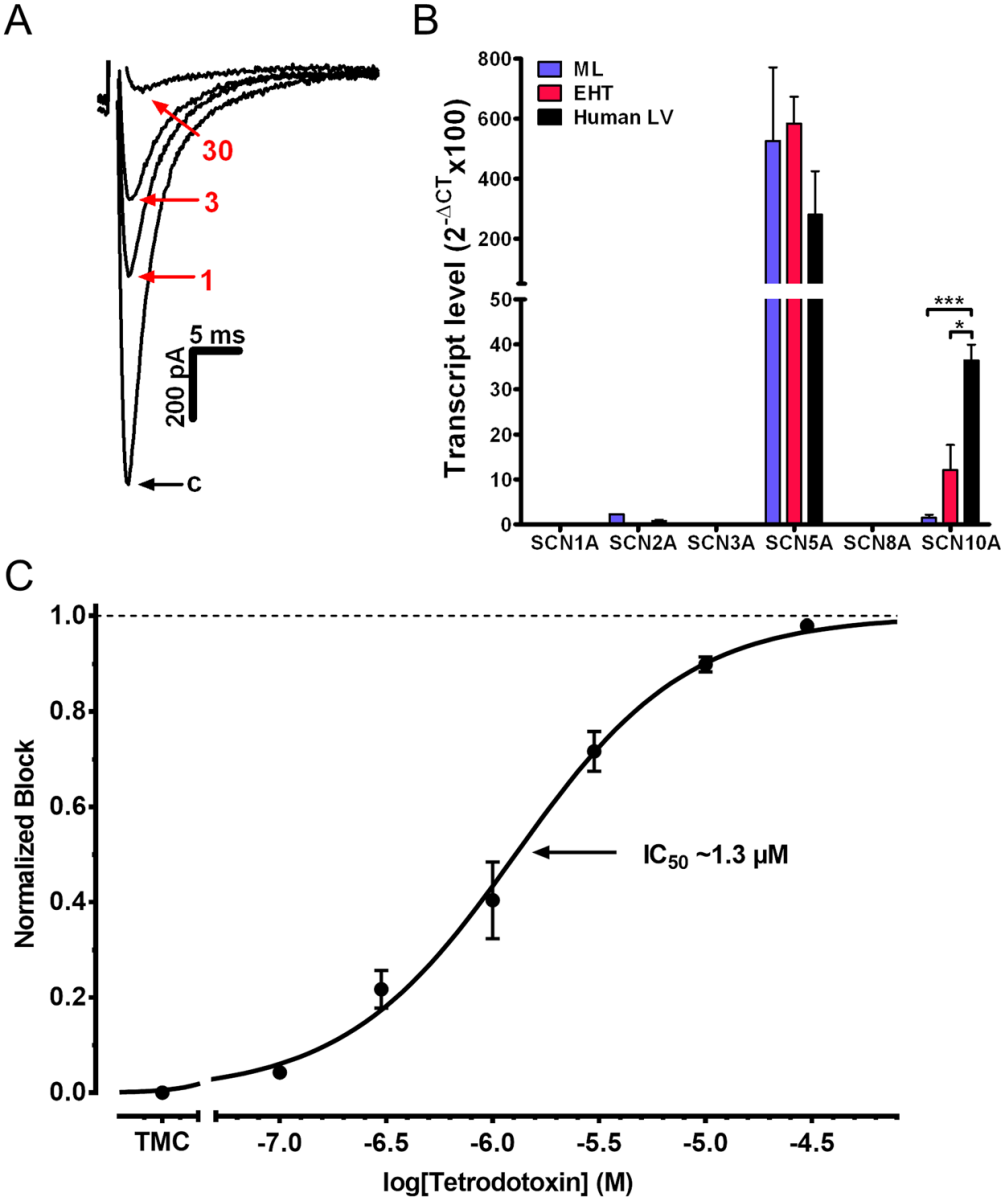
Concentration dependent effect of tetrodotoxin (TTX) on I_Na_ and expression of sodium channel subunits. (**A**) Representative original Na current tracings under control conditions and after exposure to 1, 3 and 30 µmol/L of TTX. (**B**) Transcript levels of various Na channel isoforms were quantified by qPCR in 3 samples each of human induced pluripotent stem cell-derived cardiomyocytes (hiPSC-CM) in monolayer (ML) and engineered heart tissue (EHT) format and non-failing human left ventricle (LV). SCN10A showed a different expression for ML (***p < 0.001 vs. LV) and EHT (*p < 0.05 vs. LV). Sequences of primers are provided in Supplementary Table 1. CT stands for cycle threshold of PCR amplification. (**C**) Concentration-response curve for TTX on I_Na_ in hiPSC-CM. Data are expressed as a normalized block (n = 3–9; total 30). In time-matched controls I_Na_ remains stable over several minutes (Supplementary Figure 1). IC_50_ indicates inhibitory concentration 50% of maximal response.

TTX is a Na channel blocker with high affinity for neural isoforms and low affinity for cardiac isoforms of the Na channel. We found a concentration-dependent inhibition of I_Na_ in hiPSC-CM by TTX (Fig. 3A) with a sigmoidal concentration-response relationship. A single-site binding model could be fitted to the data points (Fig. 3C). The IC_50_ was calculated at 1.3 µmol/L (95% CI 1.1 and 1.6 µmol/L) with a slope factor of 1.08. A two-site binding model did not show a better fit. Applying the extra sum-of-square-test a single-site binding model was identified as the preferred fit. Our results argue against a relevant contribution of a high affinity binding site. Accordingly, transcript levels of the low-TTX-sensitive cardiac isoform Na_V_1.5 (SCN5A) were predominant without differences between EHT, ML and non-failing human left ventricular tissue (Fig. 3B), and the TTX-resistant neuronal isoform Na_V_1.8 (SCN10A) had 8-fold (LV), 50-fold (EHT) and 350-fold (ML) lower mRNA concentrations than Na_V_1.5 (SCN5A). Transcript levels of the neuronal isoform Na_V_ 1.8 (SCN10A) were significantly lower in EHT- and ML-hiPSC-CM than in LV. Transcript levels of the highly-TTX sensitive brain-type isoforms Na_V_1.1–1.3, 1.6 (SCN1A, SCN2A, SCN3A, SCN6A) fell mainly below the cycle threshold cut-off level of 30. At the beginning of the differentiation from stem cells to cardiomyocytes SCN2A was the dominant isoform (Supplementary Figure 2). Expression levels of all isoforms described a U-shaped curve in development between day 0 and 20 of differentiation. However, at the late phase SCN5A clearly became the dominant isoform.

### Action potential measurements

**Figure 4:**
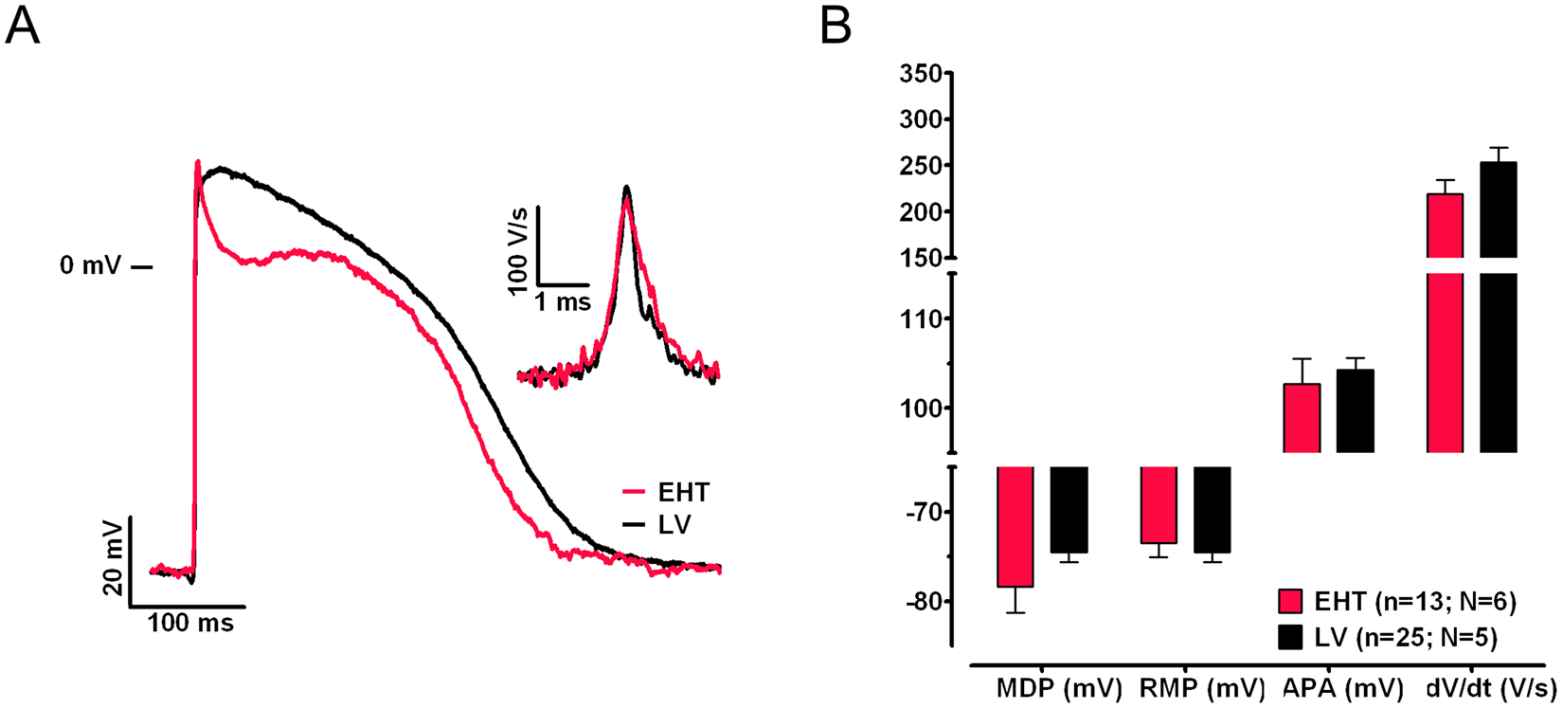
Action potential characterisation. (**A**) Example of action potentials (AP) and the AP upstroke velocity (inset) measured in human induced pluripotent stem cell-derived cardiomyocytes cultured in engineered heart tissue (EHT) or in human left ventricular tissue (LV) at 36.5 °C paced at 1 Hz. (**B**) Corresponding AP parameters. N: number of EHTs/LV tissues; n: number of impalements with the sharp microelectrode. RMP, resting membrane potential; APA, action potential amplitude; dV/dt, maximum upstroke velocity; APD_90_, AP duration at 90% repolarisation.

EHT beat spontaneously (EHT: 0.83 ± 0.12 Hz, N = 5), whereas LV tissue was quiescent. We paced EHT slightly above its intrinsic rate at 1 Hz pacing in order to compare data to LV tissue (Fig. 4) and to the literature (Table 2). AP upstroke velocity in EHT did not differ significantly from that recorded in LV tissue (EHT: 219 ± 15 V/s, n = 13, N = 6 vs. LV: 253 ± 16 V/s, n = 25, N = 5; ns; Fig. 4A and B). Maximum diastolic potential (MDP), RMP immediately before the upstroke (= take-off potential) and AP amplitude (Fig. 4B) also did not differ significantly between EHT (MDP: −78.4 ± 2.9 mV, RMP: –73.5 ± 1.6 mV, 102.7 ± 2.8 mV, n = 13, N = 6) and LV (MDP: −74.8 ± 1.1 mV, RMP: −74.8 ± 1.1 mV, APA: 104.8 ± 1.4 mV, n = 25, N = 5).

**Table 2:** Comparison of I_Na_ properties in isolated human induced pluripotent stem cell-derived and adult cardiomyocytes. HiPSC-CM: human induced pluripotent stem cell-derived cardiomyocyte; ML: monolayer; EHT: engineered heart tissue; V_0.5_: voltage of half-maximal (in)activation; k: slope factor of voltage-dependence of (in)activation; *overlap-potential (V_m_) was calculated: ((k_act_*V_0.5Inact_)−(−k_Inact_*V_0.5act_))/(k_Inact_+k_act_) (details in supplementary data); ^#^availability at overlap (%) was calculated = 1/(1+EXP^((−V0.5Inact+overlap-potential)/^k_Inact_^))^*100; I_Na_ ext: sodium concentration of the extracellular (bath) solution, I_Na_ int: sodium concentration of the intracellular (pipette) solution; MDP: maximum diastolic potential; RMP: resting membrane potential (=take-off potential); dV/dtmax: maximum upstroke velocity; APA: action potential amplitude.

CM-type	ML hiPSC	EHT hiPSC	ventri-cular	ventri-cular	atrial	atrial	atrial	atrial	atrial	atrial	atrial	ML hiPSC(on Matri-gel)	Single hiPSC (on Matri-gel)	ML hiPSC	ML hiPSC	ML hiPSC
Capacitance (pF)	24.1	27.9		194	126	73.1		72.1	66	89			17.0	42		15.8
Peak I_Na_ density (pA/pF)	−10.3	−18.5		−20.2	−17.8	−14	−30	−50.2	−37	−30.0	−160	−105	~−68	−118	−264.4	−216.7
V_0.5_ activation (mV)	−34.6	−36.2		−42.8	−38.9	−38.8		−50.2	−38.6			~−44		−42.4	−42	−34.1
k_act_	5.8	5.9		6.0	6.5			5.3	7.2					1.8		5.9
V_0.5_ inactivation (mV)	−89.8	−91.3		−97.3	−95.8	−97.1		−97.2	−95.1	−72.2	−77	−88.0	−82.8	−61.4		−72.1
k_inact_	6.1	7		5.8	5.3			6.2	7.4	4.9				7.6		5.7
∆V_0.5(Act-Inact)_ (mV)	55.2	55.1		54.5	56.9	58.3		47.0	56.5			~44		19.0		38.0
Overlap-potential (mV)*	−61.5	−61.4		−70.5	−70.2			−71.9	−66.5			~−61		−46.0		−53.4
Overlap-availability (%)^#^	0.9	1.4		1.0	0.8			1.7	2.0			~4		11.7		3.6
Days after differentiation	28	28										4–7	5–7	28	18	16
I_Na_ ext (mmol/L)	5	5		5	5	5	5	5	10	120	150	20	10	135	130	50
I_Na_ int (mmol/L)	5	5		5	5	5	5	5	10	70	10	5	5	5	5	10
Holding potential (mV)	−110	−110		−140	−140	−120	−110	−140	−140	−135	−120	−140	−120	−90	−90	−80
Temperature (°C) for I_Na_	21	21		17	17	23	21	22	22	24	21	37	22	24	36	36
Pulse frequency (Hz)	0.5	0.5		0.1	0.1	0.1	0.5		0.1	0.5	0.2					
IC_50_ TTX (µmol/L)	1.4			1.7	1.1					10.6						0.6
Temperature (°C) for AP	37	37	37				37	22	22	24	21	37	22	24	36	36
MDP (mV)		−78.4	−74.8				−72.6					−77.5	74.0	−60.9	−72.4	−75.6
RMP (=take-off) (mV)		−73.5	−74.8				−72.6					−70.5				
dV/dtmax (V/s)		219	253				230					146.5	84	13.1	115.7	27.8
APA (mV)		102.7	104.8				94.3					116	124	88.1	106.0	104.0
Author/year	This study	This study	This study	Sakak-ibara *et al*. 1993^17^, Am J Physiol	Sakak-ibara *et al*. 1992^16^, Circ Res	Li *et al*. 2009, Cardi-ovasc Res	Wettwer *et al*. 2013^7^, Cardi-ovasc Res	Bosch *et al*. 1999, Cardi-ovasc Res	Feng *et al*. 1996^21^, Am J Physiol	Schneider *et al*. 1994, Pflügers Arch Eur J Physiol	Busta-mante *et al*. 1983, Science	Herron *et al*. 2016^12^, Circ Arrhy-thmia Elect-roph	Feaster *et al*. 2015^11^, Circ Res	Ma *et al*. 2013, Int J Cardiol	Davis *et al*. 2012, Circulation	Ma *et al*. 2011, Am J Physiol Hear Circ Physiol

### Cell structure and subcellular distribution of Na_V_1.5

**Figure 5:**
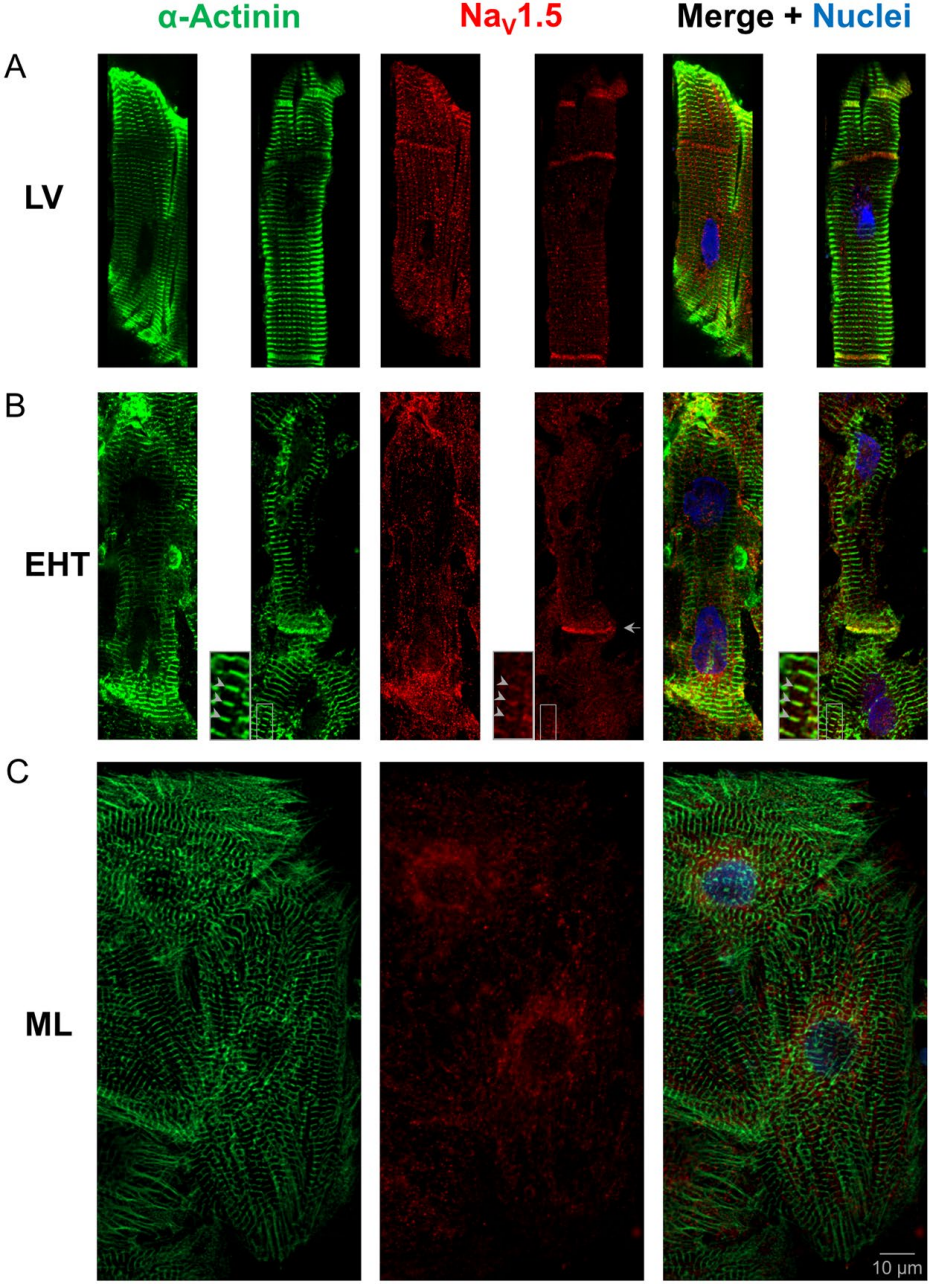
Immunofluorescence analysis. Subcellular localisation of α-actinin (green), Na_V_1.5 (red) and nuclei (blue) in a whole mount immunofluorescent confocal section of left ventricular tissue (LV, two examples, **A**) and human induced pluripotent stem cell-derived cardiomyocytes (hiPSC-CM) in engineered heart tissue (EHT, two examples, **B**) and monolayer (ML, **C**). In contrast to ML, EHT showed a parallel orientation of CM, a more rod-shaped morphology, sarcomere alignment and Na_V_1.5 enhancement at cell-cell contact (arrow). In some parts of the EHT, Na_V_1.5 was co-localised with α-actinin at Z-disks (see arrowheads in inset with 2.5 fold magnification), comparable to LV. Scale bar for all images is 10 µm. Black rectangles were placed aside confocal images in A for symmetrical appearance.

HiPSC-CM in EHT were oriented in parallel and showed a rod-shape morphology with sarcomere alignment comparable to LV tissue (Fig. 5). In contrast, hiPSC-CM in ML format showed an increased circularity and the sarcomeres were oriented in different directions even within the cell. Na_V_1.5 was distributed in the Z-disks and in the intercalated disks of adult cardiac tissue (Fig. 5). The subcellular distribution of Na_V_1.5 in ML hiPSC-CM was markedly different. It shows perinuclear enhancement with less pronounced signalling at the cell periphery and without co-localisation with α-actinin or enrichment at cell-cell contacts similar to what has been found in previous publications^18–20^. In contrast, hiPSC-CM in EHT showed a more pronounced expression of Na_V_1.5 in the periphery of the CM. Few CM in EHT showed co-localisation of Na_V_1.5 to Z-disks (arrowheads in Fig. 5B) and enhanced expression of Na_V_1.5 at cell-cell contacts orthogonal to the CM orientation (arrow in Fig. 5B), comparable to the intercalated disk of adult cardiac tissue. Proper impulse propagation does not only depend on the sodium channels, but also on polarized connexin-43 expression. We found pronounced connexin-43 staining at the cell membranes of hiPSC-CM in EHT, but no clear enhancement at end-to-end over lateral cell-cell contacts typical of adult human LV (Supplementary Figure 3).

## Discussion

In the present study, we investigated whether culture of hiPSC-CM in the EHT format leads to a higher resemblance with adult human CM in terms of I_Na_ density, upstroke velocity, CM morphology and subcellular distribution of Na_V_1.5.

I_Na_ densities are difficult to compare among different studies, as experimental conditions differ widely with respect to temperature and Na concentrations (as listed in Table 2). Here we used the same experimental conditions used previously to measure I_Na_ in human adult ventricular CM^17^, allowing for reliable comparisons with hiPSC-CM. Due to the limited availability of human adult ventricular tissue, studies analysing its electrophysiological properties are rare. To our knowledge, only one publication has studied I_Na_ in human adult ventricular CM^16^ reporting an I_Na_ density of −20.2 pA/pF. Human atrial CM I_Na_ density measured under the same conditions amounted to −17.8 pA/pF, others reported values between −4 and -30 pA/pF^7, 17, 21, 22^. Thus, the I_Na_ densities in EHT hiPSC-CM (-18.5 pA/pF) fit nicely with ventricular and atrial adult CM. The observation that I_Na_ density in EHT CM was nearly two fold higher than CM cultured in conventional ML (−10.7 pA/pF) provides evidence for the hypothesis that EHT culture improves the maturity of CM. The isometric mode of contraction of hiPSC-CM cultured on rigid surfaces (ML) in comparison to the auxotonic mode of contraction with defined load (EHT) might cause the difference in I_Na_ density. This supports recent data from hiPSC-CM cultured on soft substrate^11, 12^, suggesting that auxotonic work is essential for the development of proper I_Na_ density. I_Na_ density in mammalian CM increases constantly during cardiac development from embryonic to neonatal stages until the adult state^23–25^, which occurs in parallel with increasing SCN5A expression during differentiation and culture of hiPSC-CM (Supplementary Figure 2 and Fig. 3B). Thus, the increase in I_Na_ density might be part of the maturation process.

In order to elucidate potential mechanisms causing the greater I_Na_ density in EHT vs. ML, we reanalysed our previously published transcriptome of hiPSC-CM^15^. We focused on the expression of genes thought to influence I_Na_. On the one hand, we found lower mRNA levels in EHT for the transforming growth factor beta 1 (*TGFB1l1*) which is a multifunctional cytokine and may reduce Na_V_1.5 expression^26^. On the other hand, we found higher mRNA levels in EHT of proteins believed to enhance I_Na_ function: epidermal growth factor (*EGF*), promoting ubiquitously growth, proliferation and differentiation^27^; anchoring adaptor ankyrin-G (*ANK3*)^28^, which is involved in ion channel trafficking to the cell membrane, and plakophilin-2 (*PKP2*), which is part of cytoskeleton and cell-cell contact^29^. Interestingly, missense mutations in plakophilin-2 are known to cause arrhythmogenic right ventricular cardiomyopathy and I_Na_ deficit^30^.

The contribution of the short lasting I_Na_ to the AP also depends on inactivation kinetics, which can be characterised by fitting a single exponential non-linear curve to the negative downslope of the I_Na_. Inactivation kinetics revealed similar time constants and voltage-dependency in ML and EHT (Fig. 2B) fitting nicely to data reported for human adult CM^21^. The voltage-dependency of activation and steady-state inactivation were indistinguishable between EHT and ML (Fig. 2C), implying that the higher I_Na_ density in EHT may be explained by a greater number of functional Na channels. As experimental conditions like time after rupture and temperature are known to shift V_0.5act_ and V_0.5inact_ to approximately the same extent, we calculated the actual difference (delta) between V_0.5act_ and V_0.5inact_. We found values of 55.1 mV and 55.3 mV for EHT and ML, respectively (Table 2), which are comparable to published data^16, 17, 21^ for adult CM (54–58 mV). This finding is in contrast to previous measurements in hiPSC-CM cultured in ML showing markedly smaller values^5, 12, 22^, including a recent study that describes the culturing of hiPSC-CM on a soft substrate^12^. It is unclear if these discrepancies relate to technical differences or to intrinsic properties of different cell lines. Overall, we conclude that EHT format does not affect the voltage-dependent steady-state inactivation and activation in hiPSC-CM and these parameters are similar in our hiPSC-CM to that in human adult CM.

The difference (delta) between V_0.5act_ and V_0.5inact_ mentioned above is not trivial: more overlap should generate more window current. Previously published studies on hiPSC-CM^5, 12, 22^ have shown reduced differences (delta) between V_0.5act_ and V_0.5inact_ and as a consequence a higher calculated window current (4–12%, Table 2). Larger window currents may partly explain persistent/late I_Na_ in CM and might have a critical influence on AP duration^31^. In our experiments with hiPSC-CM, we found only a small amount of window current as in adult CM and no persistent/late I_Na_. At the maximum degree of overlap (inset Fig. 2C), I_Na_ in hiPSC-CM showed similar fractions of maximal availability and conductance (1.4% and 1.8% for EHT and ML, respectively) as human adult CM (~2% and ~1% for atrial^17^ and ventricular CM^16^, respectively). It should be noted that the amount of window current might be an additional marker in the maturation process, since window currents decrease during the development of chick embryonic CM^32^.

Interestingly, not only does the RMP influence I_Na_ density, but also vice versa, I_Na_ may influence RMP. Window I_Na_ may contribute significantly to RMP^33^, especially when inward rectifier currents are small, as in human atrial trabeculae. Imanishi *et al*. have shown that high concentrations of TTX hyperpolarized the membrane potential of quiescent human atrial trabecular by about 7 mV^33^. Consequently, window I_Na_ may be of particular relevance in hiPSC-CM, as less negative RMP and small inwardly rectifying potassium currents are consistently reported for hiPSC-CM^34^.

Sodium currents are known to recover quickly from voltage-dependent inactivation. Recovery from inactivation critically determines refractoriness and influences the susceptibility to tachyarrhythmia. In human adult atrial and ventricular CM recovery from inactivation could be fitted by a two-time constant function when plotting peak currents against different recovery time intervals^16, 17^. Earlier studies on hiPSC-CM from ML reported similar characteristics of recovery from inactivation as here^22^. It should be noted that both this study and Ma *et al*.^5^ found a somewhat faster recovery from inactivation in hiPSC-CM than in human adult CM. Whether this small difference has physiological relevance needs to be elucidated by computer modelling and functional studies.

We used TTX-inhibition of I_Na_ to evaluate whether non-cardiac isoforms contribute significantly to I_Na_ in hiPSC-CM, which might be a sign of immaturity (Supplementary Figure 2). In hiPSC-CM, the effect size for low nanomolar concentrations of TTX fits perfectly to a single-site binding model with low sensitivity (IC_50_ 1.3 µmol/L). Therefore, we would not assume relevant contribution from highly sensitive isoforms of Na channels to peak currents. Published TTX-sensitivities in atrial and ventricular CM amount to 1.1 µmol/L^17^ and 1.7 µmol/L^16^ showing that the TTX-sensitivity of I_Na_ in hiPSC-CM was similar to that in adult CM. Quantitative evaluation of the transcript levels of different Na channel isoforms confirmed that expression was dominated by the expression of the low-TTX sensitive isoform Na_V_ 1.5 (SCN5A) compared to the highly-TTX sensitive neuronal isoforms Na_V_ 1.1–1.3, 1.6 (SCN1–3A, SCN6A). The TTX-resistant neuronal isoform Na_V_1.8 (SCN10A) was expressed at intermediate levels and showed lower absolute transcription levels in hiPSC-CM than in LV.

To the best of our knowledge, we show here for the first time AP in hiPSC-CM with an upstroke velocity similar to human adult ventricular tissue (200–300 V/s). Previous studies in hiPSC-CM have reported heterogeneous and overall lower upstroke velocities (Table 2) at ~40 V/s for ventricular-like AP in isolated cells^4–6, 22^ or in embryoid bodies^9^. Recent approaches to culture hiPSC-CM on a soft substrate of extracellular matrix as single CM^11^ or ML^12^ revealed higher I_Na_ density^11, 12^ and higher upstroke velocity of 147 V/s^12^ in comparison to cultures on a stiff substrate (65 V/s). Collectively, the data indicate that culture conditions allowing auxotonic contractions of hiPSC-CM against a flexible resistance increases the resemblance in I_Na_ density known for adult CM. This will be important for the further use of hiPSC-CM in drug testing and modelling of genetically determined cardiac diseases.

We also found that AP in hiPSC-CM in EHTs showed a steeper early repolarisation and were considerably shorter than APs derived from LV tissue. A possible explanation is that APs from human tissue were all recorded from the subendocardial myocardium, which is known to express much less transient outward potassium current (I_to_) and therefore exhibits longer APs than subepicardial regions^35^. Future work is warranted to evaluate whether APs in EHT are in fact “subepicardial-like” or whether the observed difference indicate a specific hiPSC-CM phenotype or a peculiarity of the cell line under investigation.

Immunohistochemistry revealed a parallel orientation of CM with a more rod-shaped morphology and sarcomere alignment of hiPSC-CM than in ML (Fig. 5). Interestingly, the subcellular distribution of Na_V_1.5 in EHT hiPSC-CM showed an enhancement of Na_V_1.5 at cell-cell contacts and, in some CM, pronounced signals, similar to those in the intercalated disks of the LV. Since Na_V_1.5 relocates from lateral to intercalated disks during cardiac development^36^, the enhancement of Na_V_1.5 in the direction of sarcomere orientation might be another hint for structural maturation of hiPSC-CM by the EHT format. Additionally, some hiPSC-CM in EHT showed co-localisation of Na_V_1.5 with α-actinin at Z-disks, while hiPSC-CM in ML did not, as shown previously^19^.

Although the EHT format facilitated structural maturation, the cell size of hiPSC-CM was similar to those in ML format (~25 pF) and much smaller in comparison to adult LV CM (~100–200 pF)^17, 37^. As cell size increases during the embryonic development of cardiomyocytes^23^, the small cell size may indicate an early stage of development. Smaller cells have a greater membrane area to volume ratio, but the physiological relevance of this remains unclear. From a technical point of view, smaller cells should conduct smaller absolute membrane currents making patch-clamp studies more technically demanding^38^.

A limitation of this study is that all LV tissue was obtained from patients with advanced heart failure due to dilated cardiomyopathy, since access to living non-failing heart tissue is virtually impossible. We therefore cannot fully exclude electrophysiological differences to healthy tissue. However, previous publications have shown no differences in sodium current properties from failing or non-failing hearts^17^ and upstroke velocity in our hands were similar to values reported for non-failing human heart^39^.

In conclusion, we have characterized I_Na_ in hiPSC-CM in 3D EHT and conventional 2D ML culture and compared AP characteristics in hiPSC-EHTs and human ventricular tissue. The main findings are 1) a higher I_Na_ density and a similar upstroke velocity in EHT as in human adult ventricular tissue, 2) similar voltage-dependent inactivation and activation of I_Na_ in EHT and ML, 3) no evidence for relevant non-cardiac isoforms contributing to I_Na_ in hiPSC-CM and 4) a higher resemblance of hiPSC-CM in EHT to LV concerning structure and subcellular Na_V_1.5 distribution than ML. Thus, our data suggest that EHT culture of hiPSC-CM may improve the validity of *in-vitro* experiments studying electrophysiological questions.

## Methods

### An expanded method section is available in the supplementary data

#### Human materials and experimental protocols

This investigation conforms to all principles outlined by the Declaration of Helsinki and the Medical Associaton of Hamburg. According to the guidelines of the ethical review committee of the Medical Association of Hamburg, Germany, there is no need for a specific approval in this case since patient data were used anonymized. All materials from patients were taken with informed consent of the donors. Left ventricular free wall samples were obtained from patients undergoing implantation of left ventricular assist device (LVAD) or heart transplantation.

#### Generation and culture of human induced pluripotent stem cell-derived cardiomyocytes in engineered heart tissue and monolayer format

As previously described^15^, single cell suspensions of hiPSC-CM were either subjected to EHT generation in a 24-well format (1x10^6^ hiPSC-CM/EHT in a fibrin matrix (total volume 100 µl) consisting of 10 µl/100 µl Matrigel [BD Bioscience, 256235], 5 mg/ml bovine fibrinogen (200 mg/ml in NaCl 0.9% [Sigma, F4753] plus 0.5 µg/mg aprotinin [Sigma, A1153]), 2x DMEM, 10 µM Y-27632 and 3 U/ml thrombin [Biopur, BP11101104]) or cultured conventionally in ML gelatin-coated 24-well plates (4x10^5^ hiPSC-CM per well, 2 cm^2^). Culture media and duration were kept identical. For patch clamp measurements, hiPSC-CM in EHT and ML were isolated with collagenase II (200 U/ml, Worthington, LS004176) after a 24–29 day culturing period, and re-plated on gelatin-coated coverslips for 24–48 h in order to maintain adherence under perfusion.

#### Patch-clamp experiments

I_Na_ recordings were performed as described previously^7^. In brief, borosilicate glass microelectrode pipettes (tip resistances 1.5–3.0 MOhm) were used to record I_Na_ in whole-cell configuration at room temperature (21 ± 1 °C) with an Axopatch-200B amplifier (Axon Instruments, Foster City, CA). Pipette and bath solution contained 5 mmol/L NaCl.

#### Action potential measurements

APs were recorded as described previously^7^ with standard sharp microelectrodes in intact EHTs (25–60 days old) or LV tissue superfused with Tyrode´s solution at 36.5 ± 0.5 °C field-stimulated at 1 Hz (n = number of total impalements, N = number of EHT/LV tissue).

#### Immunofluorescence

Immunofluorescence was performed as described previously^15^. Briefly, EHT or LV tissue were fixed in formaldehyde overnight at 4 °C, blocked for 6 h and incubated with primary antibodies (monoclonal mouse anti-α-actinin; monoclonal rabbit anti-Na_V_1.5) and secondary antibodies and nuclear staining (Alexa Fluor® 488 goat-anti-rabbit; Alexa Fluor® 546 goat-anti-mouse; DRAQ5TM). 2D cultures were cultivated on 96-well plates and were fixed for 20 minutes at 4 °C and stained accordingly with the exception of using Hoechst 33342 for nuclei staining.

#### Statistics

GraphPad Prism 5 (GraphPad Software, San Diego, CA, USA) was used for data analyses. Curves were fitted to data points from individual experiments and all data were compared using unpaired t-tests and for groups greater than 2 One-way ANOVA followed by Tukey corrections. Two-way ANOVA was used to assess repeated measurements of current-voltage relationship (Fig. 2A). All analyses were two-tailed and a p < 0.05 was considered to be statistically significant. Group data are presented as mean ± SEM.

## Electronic supplementary material

Supplementary
