# Supplementary material for: Human iPSC-derived cardiomyocytes cultured in 3D engineered heart tissue show physiological upstroke velocity and sodium current density
Source: Sci Rep. 2017 Jul 14;7:5464. doi: 10.1038/s41598-017-05600-w (PMC5511281; doi:10.1038/s41598-017-05600-w)
Supplement: Supplementary file 1 — Supplementary [file 41598_2017_5600_MOESM1_ESM.pdf]

Supplementary data to:

Human iPSC-derived cardiomyocytes cultured in 3D engineered heart tissue show physiological upstroke velocity and sodium current density

Marc D. Lemoine[1,2,3*], Ingra Mannhardt[2,3], Kaja Breckwoldt[2,3], Maksymilian Prondzynski[2,3], Frederik Flenner[2,3], Bärbel Ulmer[2,3], Marc N. Hirt[2,3], Christiane Neuber[2,3], András Horváth[2,4], Benjamin Kloth[5], Hermann Reichenspurner[5], Stephan Willems[1,3], Arne Hansen[2,3], Thomas Eschenhagen[2,3], Torsten Christ[2,3*]

1 Department of Cardiology-Electrophysiology, University Heart Center, Hamburg, Germany

2 Department of Experimental Pharmacology and Toxicology, University Medical Center Hamburg-Eppendorf, Hamburg, Germany

3 DZHK (German Center for Cardiovascular Research), partner site Hamburg/Kiel/Lübeck, Hamburg, Germany

4 Department of Pharmacology and Pharmacotherapy, Faculty of Medicine, University of Szeged, Szeged, Hungary

5 Department of Cardiovascular Surgery, University Heart Center, Hamburg, Germany

* m.lemoine@uke.de and t.christ@uke.de

Generation and culture of human induced pluripotent stem cell-derived cardiomyocytes in engineered heart tissue and monolayer format

The undifferentiated hiPSC line C25 (kind gift from Alessandra Moretti, Munich, Germany) was expanded in FTDA medium[1] and differentiated in a three step protocol based on growth factors and a small molecule Wnt inhibitor DS07 (kind gift from Dennis Schade, Dortmund, Germany) as previously published[2]. In brief, fibrin-based EHTs were generated in agarose casting moulds with solid silicone racks in a 24-well format with $1x10^6$ hiPSC-CM in a fibrin matrix (total volume 100 µl) consisting of 10 µl/100 µl Matrigel [BD Bioscience, 256235], 5 mg/ml bovine fibrinogen (200 mg/ml in NaCl 0.9% [Sigma, F4753] plus 0.5 µg/mg aprotinin [Sigma, A1153]), 2x DMEM, 10 µM Y-27632 and 3 U/ml thrombin [Biopur, BP11101104]). For culture in conventional ML format, $4x10^5$ hiPSC-CM were plated on gelatin-coated 24-well plates. For optimal comparability, hiPSC-CM in EHT and ML were cultured under the same conditions in a 37 °C, 7% CO_2, 40% O_2 humidified cell culture incubator with the same medium consisting of DMEM (Biochrom; F0415), 10% heat-inactivated horse serum (Gibco 26050), 1% penicillin/streptomycin (Gibco 15140), insulin (10 µg/ml; Sigma I9278) and aprotinin (33 µg/ml; Sigma A1153). All experiments were performed in parallel from the same batch of cells. EHTs started coherent and stable beating at day 10-14 after casting.

Patch-clamp experiments

After a 24-29 day culturing period, hiPSC-CM in EHT and ML were isolated with collagenase II (200 U/ml, Worthington, LS004176) for 5 and 3 hours, respectively, and re-plated on gelatin-coated coverslips for 24–48 h in order to maintain adherence under perfusion[3]. 25-31 days after differentiation the hiPSC-CM were used in the patch-clamp experiments. I_{Na} recordings were performed as described previously[4]. In brief, borosilicate glass microelectrode pipettes (tip resistances 1.5–3.0 MOhm) were used to record I_{Na} in whole-cell configuration at room temperature (21±1 °C) with an Axopatch-200B amplifier (Axon Instruments, Foster City, CA). Membrane currents were low-pass filtered at 5 kHz and sampled at 10 kHz. Pipette solution contained (in mmol/L): NaCl 5, Cs methanesulfonate 90, CsCl 20, HEPES 10, Mg-ATP 4, Tris-GTP 0.4, $CaCl_2$ 3, EGTA 10 (pH 7.2, adjusted with CsOH). The bath solution contained (in mmol/L) NaCl 5, TEA Cl 120, CsCl 10, $CaCl_2$ 0.5, $MgCl_2$ 1, HEPES 10, Glucose 10 (pH 7.4, adjusted with CsOH). In order to block any remaining L-type Ca currents, 20 µmol/L nifedipine were used in all experiments. Drugs were applied using a gravity driven fast perfusion system that replaced the cell superfusate within 500 ms. Tetrodotoxin (TTX) was dissolved in a standard-bath solution over a

concentration range of 0.1–30 µmol/L (Tocris, Ellisville, MO, USA). The criteria for satisfactory voltage control for I_{Na} recordings were: smooth current–voltage (I–V) curve without abrupt jumps on the negative-slope, without threshold phenomena and with a fast capacitative transient decay.

After placement of the cover slip into the recording chamber we washed out the residual culture medium for at least 5 minutes before starting the experiment. We did not correct for offset potential. After rupture of the cell membrane a standard protocol (holding potential at –80 mV; conditioning pre-pulse to -110 mV for 1000 ms, depolarisation to -30 mV for 30 ms at a frequency of 0.5 Hz; see inset Figure 1C) was applied until I_{Na} became stable to within ~30 s. After equilibration further voltage-protocols for the I–V relationship and steady-state inactivation (at 0.5 Hz, see inset Figure 2C) and recovery of inactivation (0.2 Hz, see inset Figure 2D) were performed within 180 s post cell rupture.

Current amplitude was measured as the difference between peak inward current and current at the end of the depolarising step. Steady-state inactivation curves for I_{Na} were obtained by plotting the normalised current amplitude at the test potential as a function of the conditioning potential (V_m). A Boltzmann function was fitted to the data for each experiment and characterised using the half-maximum voltage of inactivation ($V_{0.5\ inact}$) and the corresponding slope factor (k_{inact}): $I/I_{max}=1/(1+exp^{(Vm-V0.5inact)/kinact)})$. Activation curves were calculated from I–V curves for each experiment using the equation $G=I/(V_m - E_{rev})$ (G=peak Na conductance; I=current at the test potential V_m). The apparent reversal potential E_{rev} was obtained by linear regression of two data points close to E_{rev} for each experiment. The relation between normalised peak conductance G/G_{max} and membrane potential V_m could be described by the Boltzmann equation: $G/G_{max}=1/(1+exp^{((V0.5act-Vm)/kact)})$.

Action potential measurements

Left ventricular free wall samples were obtained from patients undergoing implantation of left ventricular assist device (LVAD) or heart transplantation. All materials from patients were taken with informed consent of the donors and with approval of the local ethical boards and according to the Declaration of Helsinki. Action potentials (APs) were recorded with standard intracellular microelectrodes in intact EHTs (25-60 days after generation) or human left ventricular tissue and field-stimulated at 1 Hz (n=number of total impalements, N=number of EHT/LV tissue). Bath solution contained (in mmol/L): NaCl 127, KCl 5.4, $MgCl_2$ 1.05, $CaCl_2$ 1.8, Glucose 10, $NaHCO_3$ 22, $NaHPO_4$ 0.42, equilibrated with O_2-CO_2 [95:5] at 36.5 ± 0.5

°C, pH 7.4. APs were analysed off-line using the Lab-Chart software (ADInstruments, Spechbach, Germany).

RNA preparation and quantitative PCR

Total RNA was extracted from stem cells, hiPSC-CM, cultured either in ML or EHT-format, and non-failing human tissue using TRIzol® Reagent (Life Technologies) following manufacturer´s protocol. Stem cells were taken on day 0, 2, 4, 6, 12 and 20 of differentiation. HiPSC-CM were taken after 25-35 days of culture (ML or EHT). The conversion of cDNA was performed with SuperScript™ III First-Strand Synthesis System (Invitrogen) according to manufacturer´s instructions. For reverse transcription oligo (dT) primers supplied in the kit and 200 ng of extracted RNA were used. Use of human heart samples was reviewed and approved by the ethics committee at the University of Hamburg. QPCR was performed with SYBR-Green (Fermentas) according to manufacturer's instructions in technical triplicates. Glucuronidase-beta (GUSB) was used as the reference transcript. The target sequences were amplified during 40 cycles in an AbiPrism7000HT cycler. Primer sequences are given in Supplementary Table 1.

Immunofluorescence and immunohistochemistry

Immunofluorescence and immunohistochemistry were performed as described previously[2]. Briefly, 30-35 day old EHTs were fixed in formaldehyde overnight at 4 °C. For whole mount immunofluorescence staining, fixed EHTs were blocked (6 h in TBS 0.05 mol/L pH 7.4, 10% FCS, 1% BSA, 0.5% Triton X-100), incubated in antibody solution (TBS 0.05 mol/L pH 7.4, 1% BSA, 0.5% Triton X-100) with primary antibodies (monoclonal mouse anti-α-actinin 1:800, Sigma A7811; monoclonal rabbit anti-Nav1.5 1:200, Alomone #ASC-005), washed repeatedly with PBS, incubated in antibody solution with secondary antibodies and nuclear stainings (Alexa Fluor® 488 goat-anti-rabbit 1:800, Invitrogen; Alexa Fluor® 546 goat-anti-mouse 1:800; DRAQ5TM 1:1000, Biostatus Ltd. BOS-889-001-R050), rinsed 4-5 times in PBS and embedded in Fluoromount-G® (SouthernBiotech, 0100-01) in dented microscope slides (Carl Roth, H884.1). 2D cultures were cultivated on LDEV-free Geltrex (1:100; Gibco) coated 96-well plates (10,000 cells per well) and were fixed for 20 minutes at 4 °C and stained accordingly with the exception of using a different permeabilisation buffer (1x PBS, milk powder 3% (w/v), Triton X-100) and Hoechst 33342 (ThermoScientific) for nuclei staining. For immunohistochemistry EHT were embedded in paraffin and 3 µm thick longitudinal sections were processed for immunohistochemical staining (mouse anti-connexin-43 monoclonal antibody (BD Transduction Laboratories, 610061), dilution 1:200,

antigen retrieval: 30 min in citrate-buffer, pH 6.0. All antibodies were visualized with the multimer-technology based UltraView Universal DAB Detection Kit (Roche). All microscopic images were taken on an Axioskop 2 microscope (Zeiss).

Data analysis

The ISO2 software (MFK, Niedernhausen, Germany) was used for data acquisition and GraphPad Prism 5 (GraphPad Software, San Diego, CA, USA) was used for data analyses. Curves were fitted to data points from individual experiments and all data were compared using unpaired t-tests and for groups greater than 2 One-way ANOVA followed by Tukey corrections. Two-way ANOVA was used to assess repeated measurements of current-voltage relationship (Figure 2A). All analyses were two-tailed and a $p<0.05$ was considered to be statistically significant. Group data are presented as mean±SEM. Figures 2C and D show sigmoidal functions fitted to the mean data, which show minimal differences to $V_{0.5}$-values averaged from individual fitted experiments (Table 1).

Calculation of I_{Na} availability and overlap potential.

The potential of highest degree of overlap of steady-state inactivation and activation was calculated by equalising the corresponding Boltzmann equations(*Table 2):

$1/(1+\exp((V_{0.5act}-x)/k_{act}))=1/(1+\exp\ (V_{0.5inact}\ -x)/-k_{inact}))$

$x = ((V_{0.5inact} * k_{act}) - (-V_{0.5act} * k_{inact})) / (k_{inact} + k_{act})$.

Potential of highest overlap-degree (OP) was inserted in one of the Boltzmann equations for steady-state inactivation or activation, in order to calculate relative availability (#Table 2):

I_{Na} (%) $=1/(1+EXP^{(OP-V0.5\ act)}/k_{act}))*100$.

Supplementary Table 1. Sequences of primers for quantitative real-time PCR.

Isoform	Gene	Sequences	Product size (bp)	Reference
$Na_V1.1$	SCN1A	F 5´-CCTGGTGTTCATTGTGTTGTTC<br>R 5´-AATCGGGTGGTTTACTGTTGAG	491	[5]
$Na_V1.2$	SCN2A	F 5´-TCAAGAGAGTTCAGTGGTGCTG<br>R 5´-AGTCTCTCCGGCTGTCATTGT	418	[5]
$Na_V1.3$	SCN3A	F 5´-CTACCACATCTCCTCCTTCCT<br>R 5´-TCAGTTGACACATAGACTTTACCA	189	[5]
$Na_V1.5$	SCN5A	F 5´-CCCAGAAGCAGGATGAGAAG<br>R 5´-CGGTGAAGGTGTACTCGACA	516	[5]
$Na_V1.6$	SCN8A	F 5´-CAGCAGATGTTAGACCGACTCA<br>R 5´-CTCCTTGGCACTCTTTGAGC	155	[5]
$Na_V1.8$	SCN10A	F 5´-AGTAGAGATGGAAAATGGGCTG<br>R 5´-GTCCTGTGAAGGAAGCTCCA	327	self-designed

	Left ventricular tissue
N	5
Gender [m / f]	5/0
Age [years]	43.6±11.4
LVAD	3/5
HTX	2/5
Hypertension, n	1/5
Diabetes mellitus, n	1/5
Hyperlipidaemia, n	1/5
CAD, n	0/5
DCM	5/5
VD	1/5
LVEF [%]	20.4±2.7
Cardiovascular medication	
Digitalis	1/5
β-blockers	5/5
Ca^{2+}-channel-blockers	0
Na^{+}-channel blocker	0
Amiodarone	3/5
Diuretics	5/5
ACE-Inhibitors	4/5
AT_1-blockers	1/5
Nitrates, n	0
Lipid-lowering drugs	1/5

Supplementary Table 2. Patient characteristics belonging to the failing ventricular tissue used for experiments.

Abbreviations: AT, angiotensin receptor; CAD, coronary artery disease; DCM, dilated cardiomyopathy; HTX, heart transplantation; LA, left atrial diameter; LVEDD, left ventricular end-diastolic diameter; LVEF, left ventricular ejection fraction; VD, valve disease. Mean ± SEM.

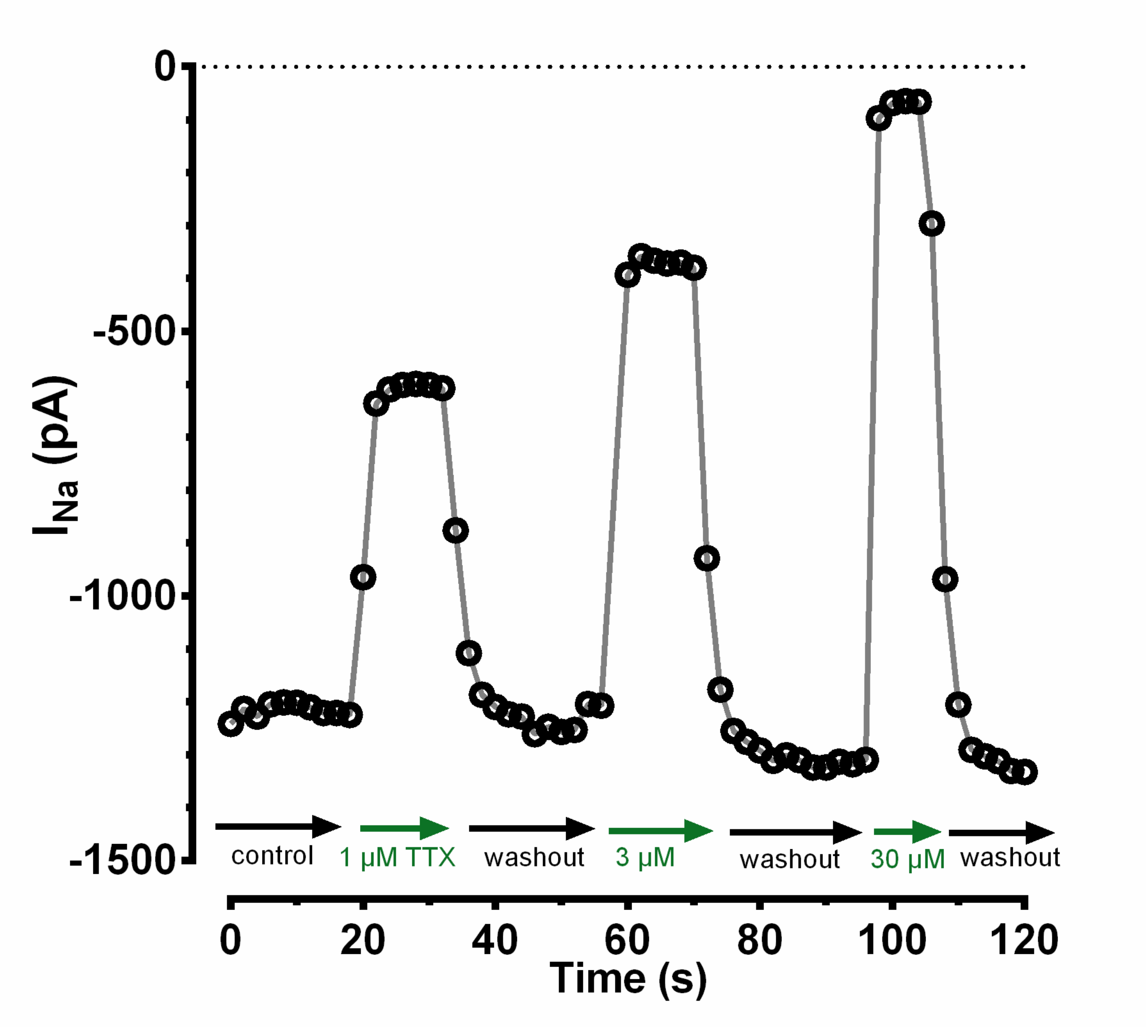

Supplementary Figure 1.
Example of time course of the peak I_{Na} upon the exposure to 1, 10 and 30 µmol/L TTX in a non-cumulative manner. Steady state was reached after ~5 s of TTX wash-in and after ~10 s of wash-out.

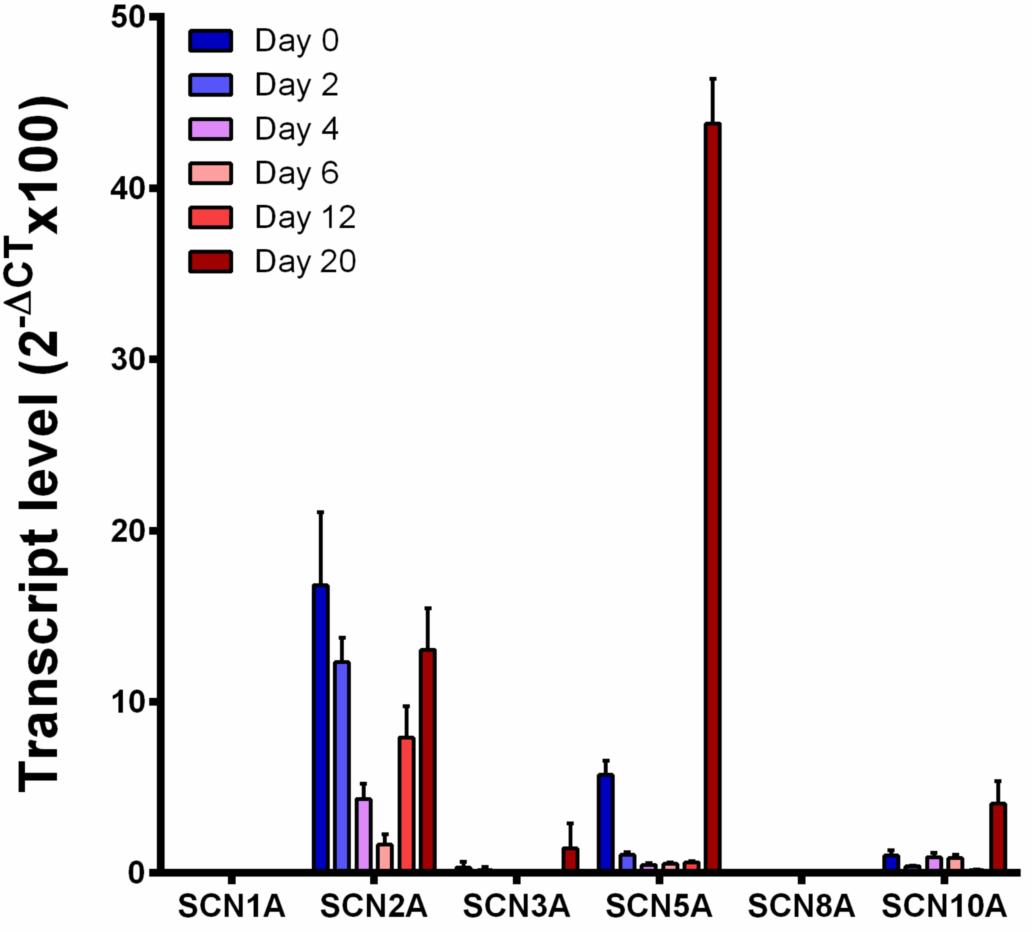

Supplementary Figure 2. Expression of Na channel isoforms during differentiation from stem cells to hiPSC-CM.

Transcript levels of various Na channel isoforms were quantified by qPCR in 3 technical replicates of stem cells during day 0 to 20 of differentiation to cardiomyocytes. Expression levels are normalized to GUSB. Sequences of primers are provided in Supplementary Table 1. CT stands for cycle threshold of PCR amplification. CT >32 cycles were defined as no expression.

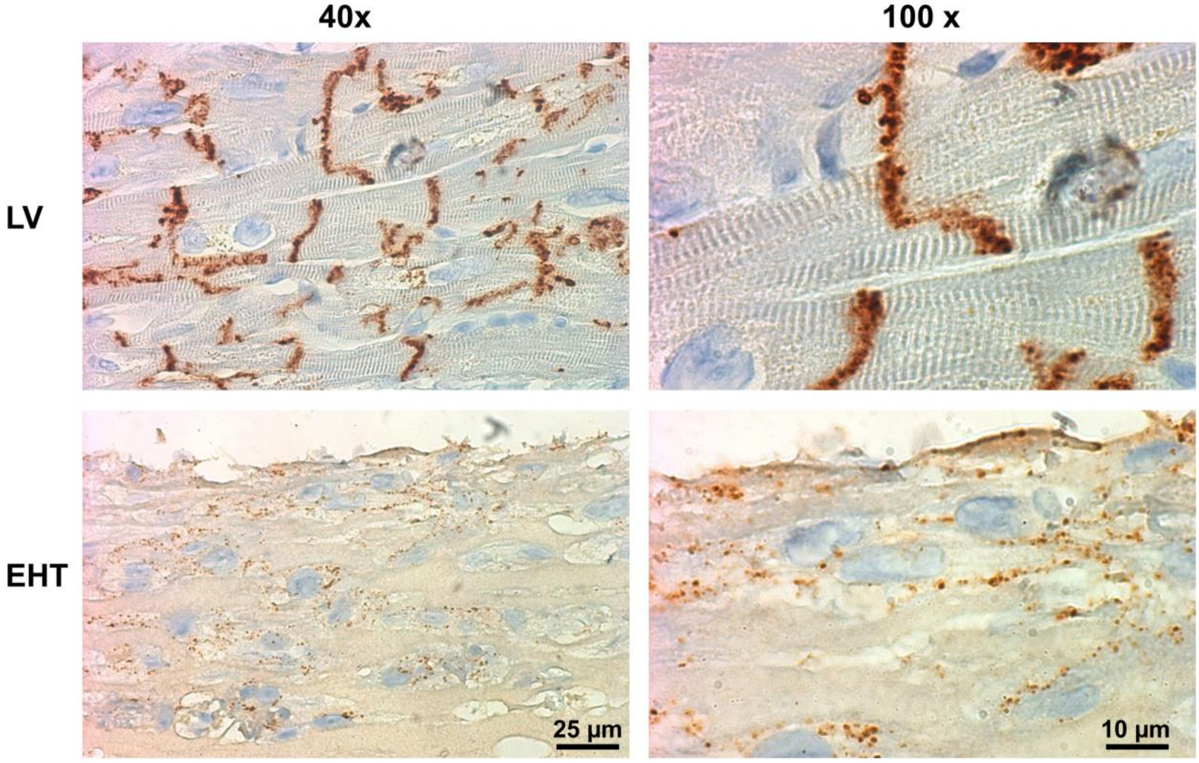

Supplementary Figure 3. Subcellular connexin-43 distribution.

Immunohistochemistry for connexin-43 (brown) staining on human adult left ventricular tissue (higher panel) and engineered heart tissue with hiPSC-CM (lower panel) with 40x (left) and 100x (right) magnification.

Supplementary References

1. Frank, S., Zhang, M., Schöler, H. R. & Greber, B. Small molecule-assisted, line-independent maintenance of human pluripotent stem cells in defined conditions. *PLoS One* **7,** (2012).
2. Mannhardt, I. *et al.* Human Engineered Heart Tissue: Analysis of Contractile Force. *Stem Cell Reports* **7,** 1–14 (2016).
3. Uzun, A. U. *et al.* Ca2+-currents in human induced pluripotent stem cell-derived cardiomyocytes effects of two different culture conditions. *Front. Pharmacol.* **7,** (2016).
4. Wettwer, E. *et al.* The new antiarrhythmic drug vernakalant: Ex vivo study of human atrial tissue from sinus rhythm and chronic atrial fibrillation. *Cardiovasc. Res.* **98,** 145–154 (2013).
5. Mishra, S. *et al.* Contribution of sodium channel neuronal isoform Nav1.1 to late sodium current in ventricular myocytes from failing hearts. *J. Physiol.* **593,** 1409–27 (2015).
